# Newly formulated, protein quality-enhanced, extruded sorghum-, cowpea-, corn-, soya-, sugar- and oil-containing fortified-blended foods lead to adequate vitamin A and iron outcomes and improved growth compared with non-extruded CSB+ in rats

**DOI:** 10.1017/jns.2017.15

**Published:** 2017-05-15

**Authors:** Nicole M. Delimont, Nicole M. Fiorentino, Alexander B. Opoku-Acheampong, Michael V. Joseph, Qingbin Guo, Sajid Alavi, Brian L. Lindshield

**Affiliations:** 1 Department of Food, Nutrition, Dietetics and Health, Kansas State University, Manhattan, KS, USA; 2 Department of Grain Science, Kansas State University, Manhattan, KS, USA

**Keywords:** Fortified blended foods, Corn–soya blends, Sorghum, Vitamin A, Iron, Protein quality, Complementary feeding, AIN, American Institute of Nutrition, CSB, corn–soya blend, DIAAS, digestible indispensable amino acid score, FBF, fortified-blended food, NRC, National Research Council, RSC, red sorghum with cowpea, SPI, soya protein isolate, USAID, United States Agency for International Development, USDA, United States Department of Agriculture, WPC, whey protein concentrate, WSC, white sorghum with cowpea

## Abstract

Corn and soyabean micronutrient-fortified-blended foods (FBF) are commonly used for food aid. Sorghum and cowpeas have been suggested as alternative commodities because they are drought tolerant, can be grown in many localities, and are not genetically modified. Change in formulation of blends may improve protein quality, vitamin A and Fe availability of FBF. The primary objective of this study was to compare protein efficiency, Fe and vitamin A availability of newly formulated extruded sorghum-, cowpea-, soya- and corn-based FBF, along with a current, non-extruded United States Agency for International Development (USAID) corn and soya blend FBF (CSB+). A second objective was to compare protein efficiency of whey protein concentrate (WPC) and soya protein isolate (SPI) containing FBF to determine whether WPC inclusion improved outcomes. Eight groups of growing rats (*n* 10) consumed two white and one red sorghum–cowpea (WSC1 + WPC, WSC2 + WPC, RSC + WPC), white sorghum–soya (WSS + WPC) and corn–soya (CSB14 + WPC) extruded WPC-containing FBF, an extruded white sorghum–cowpea with SPI (WSC1 + SPI), non-extruded CSB+, and American Institute of Nutrition (AIN)-93G, a weanling rat diet, for 4 weeks. There were no significant differences in protein efficiency, Fe or vitamin A outcomes between WPC FBF groups. The CSB+ group consumed significantly less food, gained significantly less weight, and had significantly lower energy efficiency, protein efficiency and length, compared with all other groups. Compared with WSC1 + WPC, the WSC1 + SPI FBF group had significantly lower energy efficiency, protein efficiency and weight gain. These results suggest that a variety of commodities can be used in the formulation of FBF, and that newly formulated extruded FBF are of better nutritional quality than non-extruded CSB+.

Protein–energy malnutrition, Fe and vitamin A remain some of the most common nutritional deficiencies worldwide^(^^1^^)^, and food aid targeted at improving both food security and foreign agricultural development is necessary to create sustainable and effective programmes to treat undernutrition. Fortified-blended foods (FBF) have traditionally consisted of micronutrient-fortified, partially precooked blends of milled cereals and pulses. The most commonly distributed micronutrient-fortified food aid by the United States Department of Agriculture (USDA) is corn–soya blend (CSB) FBF. Hundreds of thousands of metric tonnes of CSB are distributed annually^(^^2^^)^, and the most widely distributed is CSB+, a roasted CSB blend^(^^3^^)^. A recent report cited the importance of formulating new food aid products to improve treatment of malnutrition, which included suggestions to utilise crops that are adapted to climate change, locally available, and utilising processing methods that may destroy anti-nutritional factors thereby improving the nutritional quality of FBF^(^^2^^)^. Despite recommendations calling for new formulations, there is little research assessing nutritional outcomes from these changes compared with previous FBF formulations.

Corn, soya, sorghum and cowpea are all crops suitable for food aid due to their availability and acceptability worldwide. In 2012, The World Food Program invested 62 % of its food aid efforts to support sub-Saharan African nations^(^^4^^)^. Cowpea is a nitrogen-fixing, drought-tolerant legume that can be utilised in intercropping because it is tolerant to shade^(^^5^^–^^8^^)^, and Africa produces 96 % of global cowpea hectares^(^^5^^)^. Sorghum porridge is a widely consumed staple in many areas throughout Africa, and from 1993 to 2013, 36 % of global sorghum production came from African nations^(^^9^^,^^10^^)^. The combination of sorghum with cowpea in FBF has potential to enhance low levels of cysteine and methionine found in cowpeas, and cowpeas’ amino acid composition complements traditionally low lysine levels in sorghum^(^^5^^,^^8^^)^. Formulating new blends with sorghum and cowpea may allow for local and regional procurement aimed at improving local agricultural markets and nutritional outcomes in food aid-receiving countries^(^^2^^)^.

When consumed in large quantities, antinutritional factors such as trypsin and haemagglutinins in legumes, and phytates and tannins in cereals, may negatively affect the bioavailability of amino acids and minerals such as Zn and Fe that may contribute to stunting, wasting and micronutrient deficiencies in low-income countries^(^^11^^,^^12^^)^. Extrusion is a processing technique that has been shown to decrease antinutritional factors and improve protein and Fe bioavailability^(^^13^^–^^15^^)^ by an operation that first grinds, then partially cooks, and finally applies pressure to products to promote expansion to a desired density^(^^16^^)^. Additionally, because density of extruded products is controlled, a unique benefit of this processing is its ability to create FBF with enhanced energy and micronutrient density^(^^2^^)^. Extrusion may further benefit food aid consumers because it can create pre-cooked porridges, which take less energy and time to prepare.

In addition to suggestions to use alternative commodities and processing methods, it has been proposed that lack of animal-source protein may be a reason why FBF have not traditionally adequately prevented stunting and wasting^(^^2^^)^, although this has not been supported in a recent review^(^^17^^)^. Limitations to utilising animal protein isolates like whey protein concentrate (WPC) include: they are costly, may not provide the protein quantity to support linear growth in suggested amounts, and may lack beneficial bioactive components reported as important components of supporting growth^(^^18^^)^. A recent field trial comparing CSB+ against a complementary food product containing an animal-source protein found no difference in Fe status, or lean mass between protein-rich complementary foods in children at 6 months of age for 9 months, although there were significant improvements in knee–heel height^(^^19^^)^. Utilisation of soya-based proteins may be a safe, cost-effective and efficacious alternative to WPC^(^^20^^)^, and therefore, whey and soya protein may similarly enhance protein quality of FBF by providing amino acids that are highly bioavailable.

The primary objective in this study was to assess protein, Fe and vitamin A outcomes of newly (according to United States Agency for International Development (USAID) guidelines^(^^2^^)^) formulated extruded sorghum-, cowpea-, corn- and soya-based FBF, compared with a current, non-extruded USAID corn and soya blend FBF, CSB+. Combinations of corn, soya, sorghum and cowpea were chosen as commodities to assess whether sorghum or cowpea consumption, as recommended as alternative to corn or soya in the Food Aid Quality Report, would result in similar or better protein, vitamin A or Fe outcomes. A second aim was to compare the protein quality of a WPC-containing FBF with a soya protein isolate (SPI)-containing FBF^(^^17^^,^^18^^)^.

## Methods

### Ethical standards

We chose weanling rats, which are a well-known nutritional model, to allow for assessment of FBF protein quality, and vitamin A and Fe bioavailability during a linear growth period. The Institutional Animal Care and Use Committee at Kansas State University approved all animal procedures (protocol 3399). Welfare assessments were carried out prior to and during the experiment.

### Diets

In order to compare nutritional outcomes related to recommended formulation, six FBF were developed according to USAID food aid recommendations^(^^2^^)^, and were later reformulated to meet viscosity requirements. In reformulation, sugar replaced 15 % grain and legume flours to meet viscosity requirements and enhance acceptable sensory characteristics, and additional WPC or SPI and oil were added to meet protein and fat requirements (Table 1). The content of 15 % sugar was estimated to not exceed daily WHO guidelines, with the recommendation that no more than 50 % of energy intake come from FBF consumption assuming that the remaining energy intake would not exceed 5 % free sugar^(^^2^^)^. Vitamin and mineral premixes were formulated according to recommendations by the Food Aid Quality Report^(^^2^^)^, as 3·2 % of FBF (Research Products Company). Blends were created by extruding grain and legume flours, milling to powder, then adding sugar, vitamin and mineral premix, oil and WPC 80 % (WPC80) (Davisco Foods) or SPI 80 % (Organic Puris 1060; World Food Processing). For comparison of commodity types within FBF formulation, two white (Fontanelle 4575, 738Y), one red (217X Burgundy) sorghum with cowpea (WSC1 + WPC, WSC2 + WPC, RSC + WPC, respectively), a white sorghum (Fontanelle 4575) soya (WSS + WPC), and corn–soya blend (CSB14 + WPC), all with WPC, along with white sorghum (Fontanelle 4575) cowpea with SPI (WSC1 + SPI) extruded blends, were developed. WSC1 + WPC, WSC2 + WPC, RSC + WPC, WSS + WPC and CSB14 + WPC were formulated to compare outcomes related to consumption of different commodity types (sorghum–cowpea blends, sorghum–soya, and corn–soya). Further, CSB14 + WPC was developed to compare new formulation and extrusion of blends with a current USAID FBF (CSB+). WSC1 + SPI was formulated to compare soya with whey protein in WSC1 + WPC. CSB+ was purchased from a USDA producer (Bunge Milling), with standard preparation, which includes utilisation of heat-treated corn and soyabeans which are mixed, and micronutrient fortified. American Institute of Nutrition (AIN)-93G, which is a diet formulated to meet the National Research Council (NRC) requirements for growing rats, was included as a control diet group to facilitate assessment of adequacy of the FBF. Of note, Fe forms and concentrations, as well as vitamin A concentrations, were different between the CSB+, extruded FBF and AIN-93G. AIN-93G contained ferric citrate (6·6/100 g), while extruded FBF and CSB+ contained sodium ferric EDTA/ferrous fumarate, although at different concentrations; vitamin A concentrations in CSB+ were nearly twice those in newly formulated FBF (Table 2), and more than forty times the levels in AIN-93G. Sodium ferric EDTA was chosen to reduce mineral–antinutrient interactions found in ionised Fe forms, to improve bioavailability^(^^2^^)^. Therefore, Fe availability of FBF was expected to surpass AIN-93G (ferric citrate alone).

**Table 1.** Newly formulated extruded fortified-blended foods, corn–soya blend plus (CSB+) and American Institute of Nutrition (AIN)-93G formulations (%)*

	Sorghum flour	Cowpea flour	Soya flour	Corn flour	Sugar	Whey protein	Soya protein	Vegetable oil	Micronutrient premix
WSC1 + WPC, WSC2 + WPC, RSC + WPC	24·7	38·6	0	0	15	9·5	0	9·0	3·2
WSS + WPC	47·6	0	15·7	0	15	9·5	0	9·0	3·2
WSC1 + SPI	24·7	38·6	0	0	15	0	9·5	9·0	3·2
CSB14 + WPC	0	0	15·2	48·1	15	9·5	0	9·0	3·2

WSC1 + WPC, white sorghum–cowpea 1 with whey protein concentrate; WSC2 + WPC, white sorghum–cowpea 2 + WPC; RSC + WPC, red sorghum–cowpea + WPC; WSS + WPC, white sorghum–soya + WPC; WSC1 + SPI, WSC1 + soya protein isolate; CSB14 + WPC, corn–soya blend 14 + WPC.

* CSB+ (%): whole corn (78·4), whole roasted soya (20), vitamin mineral (0·2), tricalcium phosphate (1·16), potassium chloride (0·17). AIN-93G (%): corn starch (39·7), casein (20), maltodextrin (13·2), sucrose (10), soyabean oil (7), powdered cellulose (5), AIN-93 vitamin and mineral mix (4·5), l-cystine (0·3), choline bitartrate (0·25), *t*-butylhydroquinone (0·001).

**Table 2.** Newly formulated extruded fortified-blended foods (FBF) and corn–soya blend plus (CSB+) vitamin and mineral forticant levels (mg per 100 g)^(^^2^^)^

Newly formulated extruded FBF		CSB+	
Vitamin A palmitate	0·488	Vitamin A retinyl ester	1·04
Thiamin mononitrate (B_1_)	0·652	Thiamin mononitrate (B_1_)	0·2
Riboflavin (B_2_)	0·933	Riboflavin (B_2_)	1·4
Niacinamide (B_3_)	9·07	Niacinamide (B_3_)	8
Calcium d-pantothenate (B_5_)	3·646	Calcium d-pantothenate (B_5_)	1·6
Pyridoxine hydrochloride (B_6_)	0·752	Pyridoxine hydrochloride	1
Folic acid (B_9_)	0·087	Folic acid (B_9_)	0·11
Vitamin B_12_	0·0015	Vitamin B_12_	0·002
Vitamin D_3_	0·0292	Vitamin D_3_	0·011
Vitamin E	13·224	Vitamin E	8·3
Vitamin K	0·033	Vitamin K	0·03
Coated ascorbic acid	40·0	Coated ascorbic acid	90
Ca (tricalcium phosphate)	279·08	Ca (tricalcium phosphate)	452
Fe	13·0*	Fe	6·5*
Sodium ferric EDTA	2·0	Sodium ferric EDTA	2·5
Ferrous fumarate	11·0	Ferrous fumarate	4·0
Iodine (potassium iodide)	0·23	Iodine (potassium iodide)	0·04
Magnesium oxide	9·47		
P (tricalcium phosphate)	290·97	P (tricalcium phosphate)	290
K (potassium monophosphate)	163·19	K (potassium chloride)	140
Sodium chloride	225·67	Sodium chloride	326
Zinc sulfate	5·50	Zinc sulfate monohydrate	5

* Amount of Fe the forticant is providing.

### Fortified blended food production

Sorghum–cowpea, sorghum–soya and corn–soya flours were extruded on a single screw extruder (X-20; Wenger Manufacturing Co.). The dry feed rate was 200 kg/h for formulations made from commercially sourced flours and 166 kg/h for formulations that were obtained from flours produced from pilot milling (cowpea flour-containing FBF).

Steam and water were added in the preconditioner, where discharge temperature was maintained above 85°C, and screw speed ranged from 500 to 550 rpm. In-barrel moisture content ranged between 18 and 20 %, the die had a single circular opening of 4·1 mm. After cutting, extrudates were dried using a double-pass dryer/cooler (series 4800; Wenger Manufacturing Co.) operating at 104°C, where they were retained for 10 min, before being cooled for 5 min at room temperature. Vitamins and minerals were mixed in with other dry ingredients in steps to ensure mixing uniformity. Once dry ingredients were mixed and combined through this process, oil was added and mixed.

### Diet, macronutrient and antinutrient analysis

FBF were analysed by AOAC official methods by the University of Missouri Agricultural Chemical Laboratories. Methods included measurement for total energy (by calculation: protein = 4, carbohydrate = 4, fat = 9 kcal/g; protein = 16·7, carbohydrate = 16·7, fat = 37·7 kJ/g), protein (LECO; AOAC 990.03, 2006), fat (acid hydrolysis, 954.02, 2006), carbohydrates (by calculation: 100 % – (% crude protein + ash + crude fat + moisture)), and amino acids including available lysine (HPLC and spectrophotometry AOAC 982.30E; 975.44). Phytate and tannin contents of blends were analysed as described by Joseph^(^^21^^)^. Briefly, phytates and tannins were assessed using a Megazyme kit (Megazyme International) and methods described previously^(^^22^^)^, respectively.

### Study design

Weanling, 21- to 23-d-old male Sprague–Dawley rats (Charles River) were randomised into eight diet groups (*n* 10 per group, *n* 80 total). Animals were housed individually in wire-bottomed cages (to prevent coprophagy) with a resting board beneath food and water feeders, in a temperature-controlled facility with 12-h light and dark cycles. Rats were provided food and water *ad libitum*, fed every other day when food intake was measured, and weighed weekly for 4 weeks. Study length and size were based on the preventative prophylactic^(^^23^^)^, and protein efficiency ratio^(^^24^^)^ methods, respectively.

### Data and sample collection

At study end, rats were anaesthetised by CO_2_ inhalation, weights and lengths were recorded, and euthanised by exsanguination. Length from nose to base of tail was measured as a comparison of overall linear growth. Blood collected from cardiac puncture was divided into 2 ml EDTA-K2 vacuum tubes (Fisher) and 2 ml microcentrifuge tubes for Hb and serum, respectively. EDTA tubes were immediately placed on ice and subsequently stored at 4°C for 48 h before analysis. Blood samples in microcentrifuge tubes collected for serum analysis were allowed to rest at room temperature under Al foil to protect them from light. They were then centrifuged at 3000 ***g*** for 15 m, supernatant fraction was pipetted into microcentrifuge tubes, flash frozen in liquid N_2_, and stored at −80°C. Following blood collection, liver tissue was collected, weighed, flash frozen in liquid N_2_, and stored at −80°C. After hepatic samples were collected, bone density and total body fat mass were measured via a PIXIMUS densitometer (Lunar) following manufacturer instructions. Prior to the study, it was verified that hepatic removal had a consistent, and minimal, effect on fat mass and bone density measured.

### Iron quantification

#### Hepatic and diet iron

Hepatic Fe analysis was determined by wet ashing before quantification by flamed atomic absorption spectrometry (AAS) (Perkin Elmer AAnalyst 100). Briefly, 1 g of hepatic tissue was placed into a 50 ml acid-washed beaker, 10 ml of full-strength nitric acid was slowly added and left for 1 h for chemical decomposition. Samples were then brought to the boil, reduced to 1 ml over 2–3 h, titrated to 10 ml with deionised-distilled water, and quantified in duplicate (*n* 10) by AAS. Fe content of blends was analysed in duplicate (*n* 1) by atomic absorption spectrometry (Great Plains Analytical Laboratory AACC method 40-70.01).

#### Hb

Hb samples were prepared in triplicate (*n* 10) using Drabkin's reagent for cyanmethaemoglobin measurement (Sigma Aldrich). Samples were compared with a standard Hb curve prepared with lyophilised bovine Hb and measured by spectrophotometer at a wavelength of 540 nm according to the manufacturer's instructions.

### Retinol quantification

#### Hepatic retinol

Hepatic retinol concentrations were analysed in duplicate (*n* 10) using an adapted protocol^(^^25^^,^^26^^)^. In initial samples analysed, butylated hydroxytoluene did not protect retinol from oxidation, and was not included in the protocol. A liver sample (0·1 g) was weighed and homogenised by vortexing well with 0·25 g ascorbic acid in 5 ml ethanol^(^^26^^,^^27^^)^. Samples were placed on ice, and 1 ml of supersaturated KOH was added. After vortexing, samples were heated for 30 min in a waterbath (70°C), vortexing every 10 min for 30 s. After ensuring that tissue was totally dissolved, samples were cooled on ice for 10 min. After cooling, 6 ml of hexane were added, the sample was vortexed, the supernatant fraction was removed, and samples were dried down in a Vacufuge (Eppendorf) at 20°C. This process was repeated twice more. When approximately 1 ml of sample remained, it was vortexed for 30 s, pipetted into Eppendorf tubes, dried under N_2_, and stored at −20°C overnight (<24 h). Samples were reconstituted into 400 µl of mobile phase, vortexed well, and 20 µl were injected into the HPLC.

#### Serum retinol

Serum for all rats was pooled and prepared in duplicate (*n* 1), because of low volumes of CSB+ serum due to small body size. Pooling has been shown to be highly representative of individual serum samples *in vivo*^(^^28^^)^. Serum was extracted using a modified protocol^(^^26^^,^^29^^)^. Serum samples (150 µl) were added to an equal volume of ethanol with ascorbic acid (0·25 g/5 ml), vortexed, and extracted three times with 1 ml of hexane, with the supernatant fraction removed after each extraction. Supernatant samples were dried down under N_2_, and stored at −20°C overnight (<24 h). Samples were reconstituted into 40 µl of mobile phase, vortexed well, and 30 µl were injected into the HPLC.

#### Diet retinol

Vitamin A content of blends was analysed as described previously in duplicate^(^^30^^)^. A sample of 0·25 g of blend was weighed, transferred into a 50 ml glass centrifuge tube, then 3·5 ml of ethanol and 1·5 ml deionised-distilled water were added to the sample with 0·25 g ascorbic acid, followed by 1 ml of supersaturated KOH. FBF samples were vortexed, then placed in a 60°C waterbath for 30 min, vortexing every 10 min, then 2 ml of deionised-distilled water were added, and FBF samples were cooled on ice. Hexane (7 ml) was added, the entire sample was vortexed, the supernatant fraction was removed, and the supernatant fraction was dried down in a Vacufuge (Eppendorf) at 20°C. This process was repeated twice more. When approximately 0·5 ml of sample remained, it was vortexed for 30 s, pipetted into Eppendorf tubes, dried under N_2_, and stored at −32°C overnight (<24 h). AIN93-G, extruded FBF and CSB+ were reconstituted in 40, 80 and 160 µl, respectively, with 20 µl injected into the HPLC. Different reconstitution volumes were utilised to obtain similar retinol values, across blends with a wide range of vitamin A content.

#### Sample analysis

Samples were run on an Agilent Eclipse XDB 5 µm C_18_ (250 × 4·6 mm) analytical column at a flow rate of 1 ml/min for 20 min at 23·4°C with an autosampler (Shimadzu SIL) on an HPLC containing a LC20AB pump (Shimadzu), and a Shimadzu SPD-M20A PDA. Mobile phase consisted of 47:47:6 methanol, acetonitrile and chloroform. Samples were analysed against an external standard curve prepared using retinyl acetate (US Pharmacopeia); standards were prepared in duplicate daily from stock solutions after analysis on a spectrophotometer at 325 nm to quantify absorbance. Concentration was calculated using a molar extinction coefficient of 0·155 for retinyl acetate in ethanol^(^^31^^)^.

### Calculations

Due to differences in protein, fat, carbohydrate and total energy content between blends, as well as evidence suggesting that protein intake may not directly relate to linear growth as protein reaches a certain concentration in the diet^(^^32^^)^, energy efficiency was calculated along with protein efficiency as an indicator of protein quality:

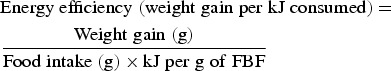


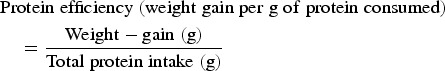


Lean mass was calculated to monitor for weight gain related to adiposity rather than linear or lean mass:

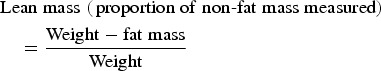


Blends were compared with digestible indispensable amino acid score (DIAAS) recommendations for protein quality assessment. DIAAS was utilised to analyse protein quality because of limitations of the protein digestibility-corrected amino acid score (PDCAAS) as an estimate of crude protein digestibility, and the recent recommendation of the FAO that DIAAS replace PDCAAS^(^^20^^)^.

### Statistical analysis

Group differences were assessed using one-way ANOVA with Tukey's test after satisfying Levene's test for homogeneity. Significance was set at *P* < 0·05; statistics were performed using SAS version 9.3 (SAS Institute, Inc.).

## Results

### Composition of fortified-blended foods

CSB+ contained 8·3 % less energy, 6·9 % more carbohydrate, 23·9 % less protein (16·4 % of composition) and 41·5 % less fat (12·1 % of composition) compared with newly formulated extruded FBF (Table 3). Lysine- and sulfur-containing amino acids did not meet DIAAS requirements for children aged 6 months to 4 years^(^^20^^)^ in CSB+ and WSC1 + SPI diets, respectively. CSB+ and AIN-93G (6·6 mg/100 g) contained 48 and 58 % less Fe than the newly formulated extruded FBF, respectively. Vitamin A content of blends was higher and lower in CSB+ and AIN-93G, respectively, compared with newly formulated extruded FBF. WPC-containing FBF groups had comparable macronutrient and micronutrient compositions (Table 3). CSB+ mean phytate content was more than three times greater than newly formulated extruded FBF (Table 3)^(^^21^^)^. There was no detectible tannin content in any of the FBF blends. Phytate content of WSC2 + WPC, RSC + WPC and WSS + WPC were similar, and more than 1·5 times greater than CSB14 + WPC; WSC1 + WPC mean phytate content was 1·2–2·5 times greater than other newly formulated blends^(^^21^^)^.

**Table 3.** Analysed macronutrient, micronutrient, and antinutrient content of fortified blended foods*

	WSC1 + WPC	WSC2 + WPC	RSC + WPC	WSC1 + SPI	WSS + WPC	CSB14 + WPC	CSB+
Total energy							
kcal/100 g	394·6	396·5	397·1	395·1	392·19	392·4	361·64
kJ/100 g	1651·0	1659·0	1661·5	1653·1	1640·9	1641·8	1513·1
Carbohydrate							
g/100 g	60·8	59·6	60·7	59·9	60·7	61·1	64·7
% energy content	61·6	60·1	61·1	60·6	61·9	62·3	71·6
Protein							
g/100 g	19·0	19·7	19·5	19·2	19·4	19·3	14·7
% energy content	19·2	19·9	19·6	19·4	19·8	19·7	16·3
Fat							
g/100 g	8·4	8·8	8·5	8·7	8·0	7·7	4·9
% energy content	19·2	20·0	19·2	20·0	18·3	18·0	12·1
Ash (g/100 g)	3·7	3·6	3·6	3·8	3·7	3·2	3·9
Crude fibre (g/100 g)	1·3	1·8	1·3	1·9	1·3	1·5	2·8
Moisture (g/100 g)	6·8	6·5	6·5	6·5	6·9	6·9	9·0
Lysine (mg/g)	74·1	70·9	72·2	60·5	69·5	68·3	52·9†
Cysteine + methionine (mg/g)	33·1	30·9	32·2	24·5†	35·0	35·7	35·3
Available lysine (mg/g)‡	72·0	67·9	68·6	58·9	67·4	66·2	52·2
Fe (mg/100 g)	15·2	15·9	15·2	16·3	15·6	15·6	8·2
Vitamin A (μg/100 g)	598·9	496·9	527·7	488·4	553·7	462·6	846·0
Phytates (mg/100 g)§	832·0	561·0	689·0	ND	557·0	318·0	1885·0
Tannins (mg/100 g)§	0·00	0·00	0·00	ND	0·00	0·00	0·00

WSC1 + WPC, white sorghum–cowpea 1 with whey protein concentrate; WSC2 + WPC, white sorghum–cowpea 2 + WPC; RSC + WPC, red sorghum–cowpea + WPC; WSC1 + SPI, WSC1 + soya protein isolate; WSS + WPC, white sorghum–soya + WPC; CSB14 + WPC, corn–soya blend 14 + WPC; CSB+, corn–soya blend plus; ND, not determined; AIN, American Institute of Nutrition.

* AIN-93G is formulated to contain 6·6 mg/100 g Fe; 23·1 µg/100 g vitamin A; macronutrient and micronutrient contents analysed in duplicate.

† Does not meet recommended mg/g amino acid content for children aged 6 months to 3 years^(^^20^^)^.

‡ By HPLC.

§ From Joseph^(^^21^^)^.

### Food intake, anthropomorphic and micronutrient outcomes

**Fig. 1.**
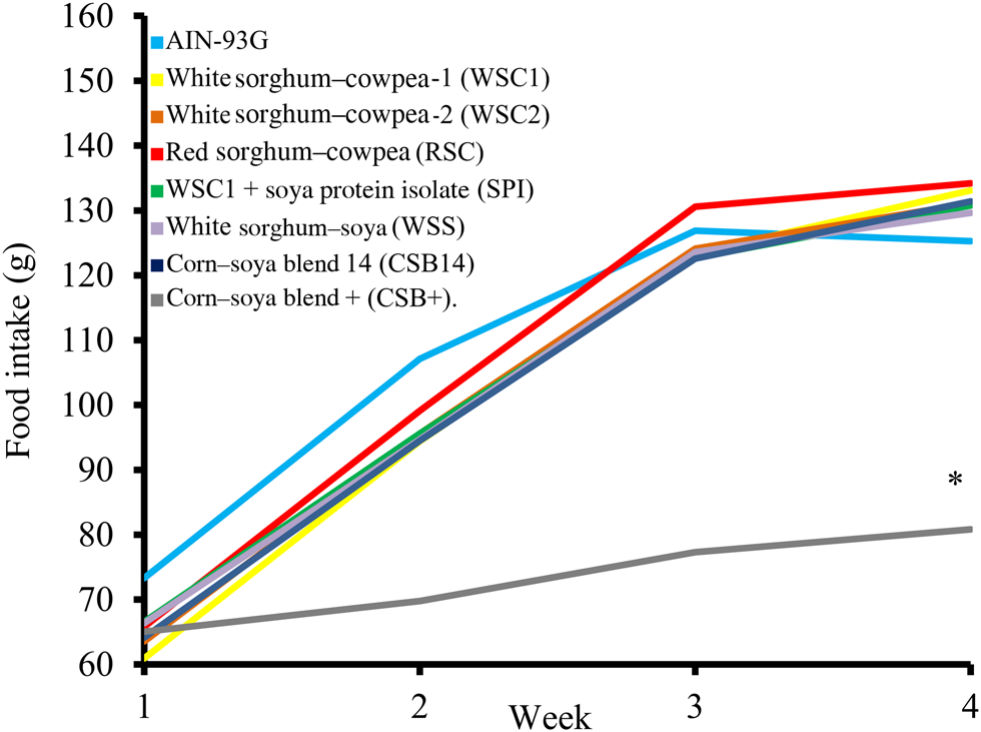
Average weekly food intake. The corn–soya plus (CSB+) group's average weekly food intake was significantly decreased during weeks 2–4 compared with extruded fortified blended food (FBF) groups and American Institute of Nutrition (AIN)-93G (*n* 10; * *P* < 0·05).

**Fig. 2.**
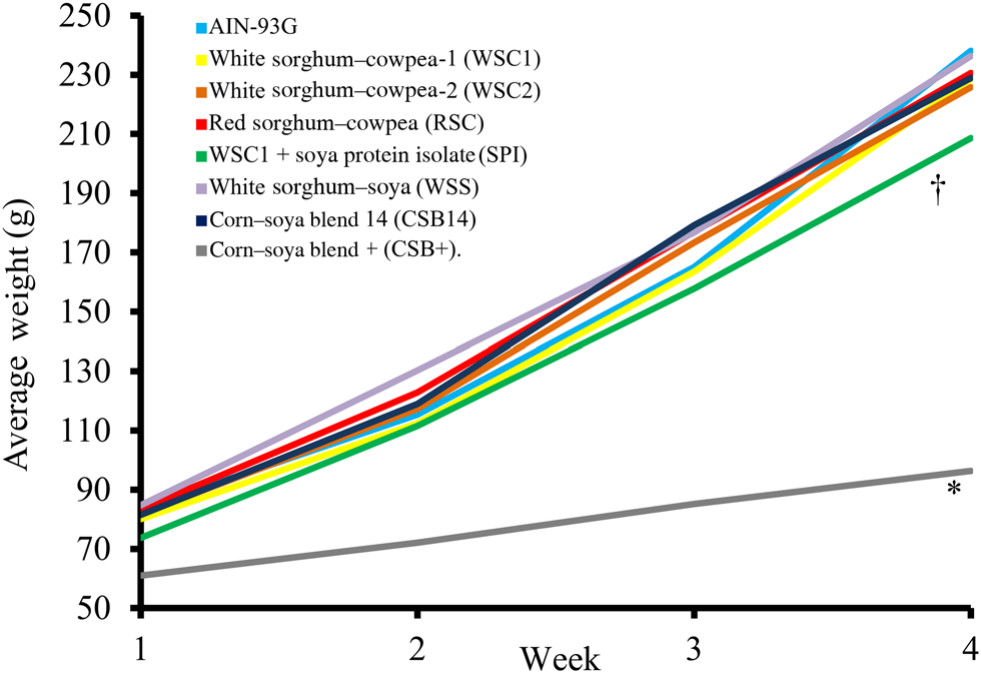
Weekly average body weights. The corn–soya plus (CSB+) group's average body weight was significantly reduced compared with extruded fortified blended food groups; the sorghum–cowpea 1 with soya protein isolate (WSC1 + SPI) group's body weight was reduced compared with American Institute of Nutrition (AIN)-93G and WSC1 with whey protein concentrate (WSC1 + WPC) (*n* 10; * *P* < 0·05 CSB+ *v*. comparison with all groups, † *P* < 0·05 WSC1 + SPI *v*. WSC1 + WPC and AIN-93G).

Food intake, weight gain, final body weights, energy efficiency, protein efficiency and linear growth changes were not significantly different between the five WPC-containing FBF groups (WSC1 + WPC, WSC2 + WPC, RSC + WPC, WSS + WPC, and CSB14 + WPC; Table 4, Figs 1 and 2). The CSB+ group's total intake was significantly reduced by 30 %, final body weight, protein efficiency were significantly decreased by greater than 50 %, energy efficiency was significantly decreased by >50 %, and length was significantly reduced by greater than 20 % compared with all groups (Table 4). During week 1, all groups consumed the same amount of FBF, while weight gain was significantly decreased (>50 %) in the CSB+-consuming group. In subsequent weeks, CSB+ consumption and growth were significantly decreased (Figs 1 and 2). Compared with the AIN-93G group, the WSC1 + SPI group gained significantly less total weight (Fig. 2). Compared with the WSC1 + WPC and AIN-93G groups, the WSC1 + SPI group had significantly lower energy and protein efficiency (Table 4).

**Table 4.** Food intake, food efficiencies and length (*n* 10) (Mean values with their standard errors)

	AIN-93G		WSC1 + WPC		WSC2 + WPC		RSC + WPC		WSC1 + SPI		WSS + WPC		CSB14 + WPC		CSB+	
	Mean	sem	Mean	sem	Mean	sem	Mean	sem	Mean	sem	Mean	sem	Mean	sem	Mean	sem
Total food intake (g)*	432·6^a^	15·7	414·3^a^	15·3	414·4^a^	11·4	429·7^a^	12·5	415·6^a^	14·8	414·3^a^	8·9	412·7^a^	9·2	293·0^b^	10·0
Total weight gained (g)†	195·9^a^	6·7	188·7^a^	6·9	184·3^a,b^	6·7	188·5^a,b^	6·9	168·9^b^	7·5	194·4^a,b^	3·2	189·0^a,b^	4·2	54·1^c^	2·6
Final body weight (g)	238·1^a^	8·0	228·5^a^	8·4	225·8^a^	7·3	230·6^a^	8·0	208·6^a^	9·1	236·3^a^	4·2	228·9^a^	4·7	96·3^b^	3·4
Energy efficiency (g/kcal x 1000)‡	113·2^a^	4·0	111·8^a^	1·9	109·1^a,b^	2·9	107·6^a,b^	2·5	101·3^b^	1·8	114·7^a^	1·4	113·3^a^	1·6	49·0^c^	2·2
Energy efficiency (g/kJ x 1000)‡	27·1^a^	1·0	26·7^a^	0·5	26·1^a,b^	0·7	25·7^a,b^	0·6	24·2^b^	0·4	27·4^a^	0·3	27·1^a^	0·4	11·7^c^	0·5
Protein efficiency (g/g)§	2·40^a^	0·08	2·40^a^	0·04	2·22^a,b^	0·06	2·19^a,b^	0·05	2·14^b^	0·04	2·35^a,b^	0·28	2·29^a,b^	0·03	1·16^c^	0·17
Length (cm)	21·8^a^	2·3	21·6^a^	0·3	21·4^a^	0·3	21·5^a^	3·2	20·7^a^	3·1	21·8^a^	0·2	21·9^a^	2·3	16·8^b^	0·4

AIN, American Institute of Nutrition; WSC1 + WPC, white sorghum–cowpea 1 with whey protein concentrate; WSC2 + WPC, white sorghum–cowpea 2 + WPC; RSC + WPC, red sorghum–cowpea + WPC; WSC1 + SPI, WSC1 + soya protein isolate; WSS + WPC, white sorghum–soya + WPC; CSB14 + WPC, corn–soya blend 14 + WPC; CSB+, corn–soya blend plus.

a,b,c Mean values with unlike superscript letters were significantly different (*P* < 0·05).

* Food intake: measured every other day by subtracting food remaining from food given (g).

† Total weight gained: cumulative weight gain from weeks 1 to 4 (g).

‡ Energy efficiency: total weight gain (g) divided by total energy (kcal or kJ) consumed.

§ Protein efficiency: total weight gained (g) divided by total protein consumed (g).

There were no significant differences in lean mass, bone mineral density, Hb, hepatic Fe, serum retinol or hepatic Fe in newly formulated extruded FBF groups. Bone mineral density was significantly lower in the CSB+ group compared with the AIN-93G group. Liver weight as a percentage of body mass was significantly lower in the CSB+ group compared with all groups (Table 5). The AIN-93G group had significantly, and WSS+WPC non-significantly, lower hepatic Fe levels compared with the remaining groups (Table 6). WSC2 + WPC, RSC + WPC and WSC1 + SPI groups had significantly lower Hb levels than the CSB+ group (Table 6). Serum retinol levels were not significantly different between groups, while CSB+ and AIN-93G had significantly higher and lower hepatic retinol concentrations than all groups, respectively (Table 6).

**Table 5.** Anthropometric outcomes (Mean values with their standard errors)

	AIN-93G		WSC1 + WPC		WSC2 + WPC		RSC + WPC		WSC1 + SPI		WSS + WPC		CSB14 + WPC		CSB+	
	Mean	sem	Mean	sem	Mean	sem	Mean	sem	Mean	sem	Mean	sem	Mean	sem	Mean	sem
Lean mass (%)*	89·9^a^	0·5	90·0^a^	0·4	89·6^a^	0·3	89·7^a^	0·4	88·1^a^	0·6	90·3^a^	0·3	89·5^a^	0·5	90·4^a^	0·5
Bone mineral density (g/cm^2^) × 1000	87·4^a^	3·1	80·4^a,b^	2·2	78·3^a,b^	1·7	82·0^a,b^	2·5	77·3^a,b^	1·0	79·0^a,b^	1·3	80·7^a,b^	4·6	73·7^b^	1·6
Liver weight/body weight (%)†	5·63^a^	0·2	5·46^a^	0·1	5·50^a^	0·2	5·47^a^	0·2	4·90^a^	0·2	5·58^a^	0·2	5·73^a^	0·2	4·00^b^	0·1

AIN, American Institute of Nutrition; WSC1 + WPC, white sorghum–cowpea 1 with whey protein concentrate; WSC2 + WPC, white sorghum–cowpea 2 + WPC; RSC + WPC, red sorghum–cowpea + WPC; WSC1 + SPI, WSC1 + soya protein isolate; WSS + WPC, white sorghum–soya + WPC; CSB14 + WPC, corn–soya blend 14 + WPC; CSB+, corn–soya blend plus.

a,b Mean values with unlike superscript letters were significantly different (*P* < 0·05).

* Lean mass: total weight minus fat mass and divided by total weight × 100.

† Liver weight/body weight %: liver weight divided by body weight × 100.

**Table 6.** Circulating and hepatic iron and vitamin A levels (Mean values with their standard errors)

	AIN-93G		WSC1 + WPC		WSC2 + WPC		RSC + WPC		WSC1 + SPI		WSS + WPC		CSB14 + WPC		CSB+	
	Mean	sem	Mean	sem	Mean	sem	Mean	sem	Mean	sem	Mean	sem	Mean	sem	Mean	sem
Hb (g/l)	186^a,b^	6	179^a,b^	5	177^a^	9	179^a^	6	173^a^	5	183^a,b^	4	187^a,b^	9	202^b^	4
Hepatic Fe (μmol/g) × 10	14·2^a^	1·7	29·3^b^	2·9	27·2^b^	3·0	30·0^b^	2·7	27·8^b^	2·8	20·7^a,b^	2·9	27·2^b^	3·2	31·0^b^	2·5
Serum retinol (ng/μl)	89·6^a^	3·5	73·9^a^	11·2	74·5^a^	2·2	77·9^a^	2·7	82·6^a^	1·8	71·3^a^	1·2	67·4^a^	10·1	55·7^a^	5·3
Hepatic retinol (ng/mg)	78·1^a^	5·0	479·4^b^	17·8	473·8^b^	27·8	482·5^b^	20·4	585·5^b^	28·6	423·8^b^	19·4	460·9^b^	28·9	1478·8^c^	95·2

AIN, American Institute of Nutrition; WSC1 + WPC, white sorghum–cowpea 1 with whey protein concentrate; WSC2 + WPC, white sorghum–cowpea 2 + WPC; RSC + WPC, red sorghum–cowpea + WPC; WSC1 + SPI, WSC1 + soya protein isolate; WSS + WPC, white sorghum–soya + WPC; CSB14 + WPC, corn–soya blend 14 + WPC; CSB+, corn–soya blend plus.

a,b,c Mean values with unlike superscript letters were significantly different (*P* < 0·05).

### Comparing fortified-blended foods with National Research Council recommendations

Due to significantly different anthropometric outcomes, WSC1 + SPI and CSB+ macronutrient and micronutrient contents were compared with NRC recommendations for growing rodents^(^^33^^)^. WSC1 + WPC content is also included as a representative WPC-containing FBF given its similarity in formulation to WSC1 + SPI. Comparing WSC1 + WPC composition with the NRC recommendations assisted in identifying composition differences that may have contributed to significant outcomes observed in the CSB+ and WSC1 + SPI groups. Micronutrient content of CSB+ and WSC1 + SPI met or exceeded recommended requirements for weaning rodents with the exception of vitamin B_12_, folic acid and vitamin K, which were not different from WSC-1 + WPC, whose growth was not suppressed compared with control (Table 7). CSB+ thiamine content did not compare to WSC1 + WPC or meet NRC requirements. CSB+ and WSC1 + SPI levels were below requirements for sulfur-containing amino acids (53 and 48 % of recommendation, respectively), and CSB+ lysine concentration was 15·2 % less than requirement. WSC1 + WPC met all NRC recommendations^(^^33^^)^.

**Table 7.** Comparison of National Research Council (NRC)^(^^33^^)^ growing rodent dietary needs *v*. formulation per 100 g of corn–soya blend plus (CSB+), white sorghum–cowpea 1 with soya protein isolate (WSC1 + SPI) and white sorghum–cowpea 1 with whey protein concentrate (WSC1+WPC)

	NRC	CSB+	WSC1 + SPI	WSC1 + WPC		NRC	CSB+	WSC1 + SPI	WSC1 + WPC
Vitamin A (μg)	70	1038	488	488	Total fat (g)	5	4·88	8·74	8·37
Vitamin D_3_ (μg)	2·5	11·04	29·2	29·2	Protein (g)	15	14·74	18·53	19·02
Vitamin E (mg)	1·8	8·3	13·2	13·2	Arginine (g)	0·43	0·93	1·33	0·99
Vitamin K (μg)	100	30	33	33	Aromatic amino acids (g)*	1·02	1·17	1·21	1·42
Thiamin (mg)	0·4	0·2	0·652	0·652	Histidine (g)	0·28	0·41	0·53	0·49
Riboflavin (mg)	0·3	1·4	0·933	0·933	Isoleucine (g)	0·62	0·61	0·87	0·98
Vitamin B_6_ (mg)	0·6	1	0·752	0·752	Leucine (g)	1·07	1·34	1·59	1·82
Pantothenic acid (mg)	1	1·6	3·646	3·646	Lysine (g)	0·92	0·78	1·16	1·41
Folic acid (μg)	100	110	87	87	Methionine + cysteine (g)	0·98	0·52	0·47	0·63
Niacin (mg)	1·5	8	9·07	9·07	Threonine (g)	0·62	0·54	0·68	0·94
Vitamin B_12_ (μg)	5	2	1·5	1·5	Tryptophan (g)	0·2	0·18	0·25	0·28
Iodine (μg)	15	40	23	23	Valine (g)	0·74	0·74	0·98	1·04
Total Fe (mg)	3·5	6·5	13	13					
Zn (mg)	1·2	5	5·5	5·5					

* Aromatic amino acids: phenylalanine, tyrosine, tryptophan.

## Discussion

In this study, consumption of newly formulated, protein quality-enhanced blends resulted in improved protein efficiency, and vitamin A and Fe availability outcomes compared with a current FBF (CSB+) and a control diet formulated for growing rats regardless of cereal or legume combination. Further, there were no differences in protein efficiency, vitamin A and Fe outcomes among newly formulated extruded FBF. This suggests that cowpea- and sorghum-based FBF support protein, vitamin A and Fe outcomes as effectively as corn and soya in developed blends.

CSB+ consumption resulted in poor growth outcomes, suggesting poor protein quality in the blend. The CSB+ group consumed less FBF, had weight and length suppression, and lower energy and protein efficiency compared with all groups. The SPI-containing FBF-consuming group also had significantly lower energy efficiency, protein efficiency and weight gain compared with a similar FBF group with WPC. Adiposity did not differ between FBF groups, micronutrient outcomes were similar among extruded FBF; however, the CSB+ group's vitamin A and Fe hepatic levels were significantly greater than other groups.

Several factors probably led to changes in growth observed in the CSB+ group, and to a lesser extent, the SPI-consuming group. Certainly, significant reduction in CSB+ consumption contributed to growth suppression, but growth was inhibited with similar food intake to other groups from week 1. Although reductions in growth were seen in the first week of feeding, when intake was consistent with other groups, CSB+ intake in subsequent weeks was significantly less than all other groups. Given growth issues despite similar food intake in the first week of feeding, food quality issues should be considered. Blends met requirements of total protein and fat intake when compared with NRC recommendations for rodents^(^^33^^)^; however, selected amino acids were lower than recommendations, including methionine + cysteine (WSC1 + SPI and CSB+) and lysine (CSB+, Table 7). While severe limitations in lysine may reduce rodent growth, it was probably not the only cause of growth restriction in the CSB+-consuming group. For example, up to 50 % of lysine recommendations in feed did not reduce growth in 6-week-old Sprague–Dawley rats^(^^34^^)^. Relative deficiency of methionine is a well-known growth inhibitor in weanling rats^(^^35^^)^, but given that the methionine content was lower in WSC1 + SPI than CSB+, it is unlikely that lack of methionine was the cause of observed growth suppression. These findings may, however, explain the small but significant decreases in weight gain and energy efficiency in the WSC1 + SPI group compared with the WSC1 + WPC group. Some of the growth impairment in the CSB+ group may have been due to several limiting amino acids (methionine, cysteine, lysine, leucine and tryptophan) or, more likely, reduction in protein and starch digestibility. The NRC recommends that protein sources be ‘high quality’^(^^33^^)^, and while protein content may have been adequate, protein digestibility may have been poor in the CSB+ group compared with the newly formulated extruded FBF. One noteworthy consideration is that CSB+ is partially cooked, but its preparation requires boiling to complete cooking, while extruded blends are considered completely cooked. Complete cooking improves starch and protein digestibility, supported by multiple observations that extrusion improves cereal and legume amino acid digestibility^(^^36^^–^^38^^)^. It is important to note that lack of extrusion, and reduced digestibility in CSB+ may have caused reductions in overall feeding by rats during the study duration. Perhaps more importantly, phytate content of blends was greatly reduced in newly formulated extruded blends, potentially due to extrusion, and reformulation of extruded FBF with less grain and legume by volume. CSB+ phytate content was more than three times the levels found in newly formulated extruded blends^(^^21^^)^, and inhibition of growth may be attributed in part to reductions in amino acid bioavailability and enzyme activity of dietary and mucosal proteins, found *in vivo* with consumption of phytate-containing foods^(^^39^^)^.

In the SPI-consuming group, it is possible that reduced protein digestibility when compared with WPC may have accounted for the small decrease (10 %) in weight gain. Whether outcomes in this study would result in differences in children consuming a varied diet remains to be seen. For example, no differences in growth were found in 6- to 12-month-old infants consuming soya, casein or rice formula along with complementary feeding^(^^40^^)^. In infant studies, plant protein sources have been as efficacious as other ready-to-use foods for growth^(^^41^^,^^42^^)^, despite possibly poor protein quality identified in our study. Dietary variety beyond food aid may contribute to these findings as well. A 2014 review found that FBF containing isoenergetic, isonitrogenous sources of animal-source proteins did not enhance linear growth compared with plant proteins, suggesting that animal protein itself may not be needed to be included in FBF^(^^17^^)^. While total weight gained is used as a surrogate for protein efficiency, studies have also supported that weight gain is not an accurate surrogate for prevention of stunting, more accurately depicted by linear growth. Despite reduced protein efficiency and weight gain, our study supports that a WPC-containing FBF did not significantly enhance linear growth compared with an SPI-containing FBF in rats. Given the higher cost of WPC when compared with SPI, it may be prudent to further explore the use of SPI, or other high-quality plant protein sources within FBF.

It is possible that animal feeding behaviours had an effect on growth in the CSB+ group. One possibility is that the CSB+ group did not consume CSB+ as well as other FBF groups because they contained sugar. Given that lean mass and food intake were unchanged in newly formulated extruded FBF groups compared with the AIN-93G group, which also did not contain sugar, it is unlikely that sugar led to overeating of blends. It is possible that sugar enhanced taste, or masked unappealing flavours of the extruded FBF. For example, some studies have cited an improved taste of corn and soya blended foods with enhanced sweetness^(^^43^^)^.

Similarities in the micronutrient outcomes make it unlikely that micronutrient differences were responsible for the observed growth suppression. While extrusion has been demonstrated to enhance micronutrient bioavailability^(^^44^^,^^45^^)^, the combination of higher levels of vitamin A in CSB+ and subsequently less demand for micronutrients due to slower growth rates probably resulted in the elevated hepatic Fe and retinol levels observed. Additionally, animals in the CSB+ group did not show overt signs of other micronutrient deficiencies, and their livers were not enlarged compared with other groups. Given that circulating retinol and Hb differences were not observed among groups, vitamin A toxicity or Fe toxicity were also unlikely causes for growth suppression.

One interesting outcome was the decrease in the WSS + WPC group's hepatic Fe levels. Compared with other sorghum-containing groups, WSS + WPC contained 23 % more sorghum, and, despite no differences in tannin or phytic acid content among blends, higher sorghum composition may explain this downward trend in hepatic Fe levels. One study found that in mice fed rice, wheat, millet or sorghum, Fe was most poorly absorbed from sorghum compared with all other grain types^(^^46^^)^. Despite these findings, given the non-significant relationship between hepatic Fe and grain types, it may be most important to consider availability, cost and preference of consumers of these products rather than small changes in biochemical markers when selecting commodities for FBF.

Interestingly, our findings do not support further differences in protein quality or in biochemical markers with the consumption of newly formulated blends containing varying levels of antinutritional factors regardless of grain type. For example, WSC1 + WPC contained more phytates than the other sorghum–cowpea formulations, but had similar energy and protein efficiency, and micronutrient outcomes. Our results may suggest that differences in digestibility and bioavailability of nutrients in sorghum and cowpea may be negated by reductions in antinutritional factors. Further, lack of differences in outcomes between groups consuming newly formulated extruded blends regardless of phytate level may suggest possible threshold, or dose-mediated adaptation, suggested previously^(^^47^^–^^49^^)^. Long-term studies exploring protein and micronutrient adaptation in humans may enhance understanding of FBF quality and efficacy during different lifecycle stages.

### Limitations

Given consumption level and composition differences, it is not possible to specifically identify factors that contributed to the inhibition of CSB+ growth outcomes compared with other groups. FBF were consumed as dry powders rather than cooked porridges. Sorghum protein digestibility has been reported to decrease after cooking in water^(^^50^^)^, although our blends are cooked during extrusion, and it is possible that protein digestibility would not be decreased with addition of hot water during their preparation. Lack of cooking may have contributed to poor protein and starch digestibility of CSB+. Further, lack of cooking limits generalisability of this study to human consumption, where blends would be consumed as porridge. We did not obtain antinutritional information for WSC1 + SPI, so our interpretation of findings in this group is limited. Newly formulated extruded FBF prepared porridges contain increased solids when compared with CSB+ (20 and 13·79 %, respectively), which is not a difference we were able to assess in this study. This study was limited to a rapid growth period, but did not follow animals through transitions into later life. This limits the ability to ascertain whether newly formulated extruded FBF support long-term growth. Additionally, the study was limited to FBF consumption only, rather than ‘complementary’ consumption along with other food items.

### Conclusions

These results suggest that a variety of commodities can be used in extruded FBF newly formulated with high-quality protein, sugar and oil, which are of better nutritional quality than CSB+. Further studies that compare prepared FBF porridges to gain a better understanding of poor growth outcomes in the CSB+ group are warranted. Given the potential cost savings of using plant protein sources, further research comparing soya, or other plant proteins, *v*. whey protein in FBF is warranted. A field trial is currently assessing the efficacy of these newly formulated extruded porridges in combating micronutrient deficiencies and supporting linear growth in children.
